# IMGT^®^ Biocuration and Comparative Analysis of *Bos taurus* and *Ovis aries* TRA/TRD Loci

**DOI:** 10.3390/genes12010030

**Published:** 2020-12-28

**Authors:** Perrine Pégorier, Morgane Bertignac, Viviane Nguefack Ngoune, Géraldine Folch, Joumana Jabado-Michaloud, Véronique Giudicelli, Patrice Duroux, Marie-Paule Lefranc, Sofia Kossida

**Affiliations:** IMGT®, The International ImMunoGeneTics Information System®, Institut de Génétique Humaine (IGH), Centre National de la Recherche Scientifique (CNRS), Université de Montpellier (UM), 34000 Montpellier, France; perrine.pegorier@igh.cnrs.fr (P.P.); morgane.bertignac@igh.cnrs.fr (M.B.); viviane.nguefack-ngoune@igh.cnrs.fr (V.N.N.); geraldine.folch@igh.cnrs.fr (G.F.); joumana.michaloud@igh.cnrs.fr (J.J.-M.); veronique.giudicelli@igh.cnrs.fr (V.G.); patrice.duroux@igh.cnrs.fr (P.D.); Marie-Paule.Lefranc@igh.cnrs.fr (M.-P.L.)

**Keywords:** IMGT, immunoinformatics, immunogenetics, T cell receptor, TRA locus, TRD locus, *Bos taurus*, *Ovis aries*

## Abstract

The adaptive immune response provides the vertebrate immune system with the ability to recognize and remember specific pathogens to generate immunity, and mount stronger attacks each time the pathogen is encountered. T cell receptors are the antigen receptors of the adaptive immune response expressed by T cells, which specifically recognize processed antigens, presented as peptides by the highly polymorphic major histocompatibility (MH) proteins. T cell receptors (TR) are divided into two groups, αβ and γδ, which express distinct TR containing either α and β, or γ and δ chains, respectively. The TRα locus (TRA) and TRδ locus (TRD) of bovine (*Bos taurus*) and the sheep (*Ovis aries*) have recently been described and annotated by IMGT^®^ biocurators. The aim of the present study is to present the results of the biocuration and to compare the genes of the TRA/TRD loci among these ruminant species based on the *Homo sapiens* repertoire. The comparative analysis shows similarities but also differences, including the fact that these two species have a TRA/TRD locus about three times larger than that of humans and therefore have many more genes which may demonstrate duplications and/or deletions during evolution.

## 1. Introduction

The adaptive immune response arose in jawed vertebrates or gnathostomata more than 450 million years ago. It is characterized by the remarkable specificity and the extreme diversity of their antigen receptors [1]. These antigen receptors of the adaptive immune response are the immunoglobulins (IG) or antibodies of the B cells and plasmocytes [2], and the T cell receptors (TR) of the T cells [3]. The IG recognize antigens in their native form, whereas the TR recognize processed antigens, which are presented as peptides by the major histocompatibility (MH) proteins.

T cell receptors (TR) are divided into two groups, αβ and γδ, which express distinct TR containing either α and β, or γ and δ chains, respectively [3]. Each TR chain comprises a variable and a constant domain. The variable domain is the result of one rearrangement between variable (V) and joining (J) genes for α and γ chains, and two consecutive rearrangements between diversity (D) and J genes then between V and partially rearranged D-J genes for β and δ chains. After transcription, the V–(D)–J sequence is spliced to the constant (C) gene to give the final transcript [3].

The human TRα (TRA) locus consists of a cluster of 56 TRAV genes located upstream (in 5${}^{\prime }$) of a J-C cluster, composed of sixty-one TRAJ and one TRAC [3]. The TRδ (TRD) locus is nested in the TRA locus between the TRAV and the TRAJ genes [3]. This locus comprises a cluster of one TRDV, three TRDD, four TRDJ, one TRDC and another TRDV, TRDV3, in inverted transcriptional orientation downstream of TRDC. One TRDV gene is also present among the TRAV cluster. The particularity of this locus is that some V genes rearrange to both TRAJ and TRDD-TRDJ genes [3].

Animal species, such as mice and larger animals, are essential models for biological research and studies on farm animals, and contribute, for example, to fundamental and applied immunology [4]. Furthermore, several veterinary species are useful for biotechnological applications that can also be applied to human medicine. This justifies the interest of scientists in the locus genomic organization of IG and TR genes involved in the adaptive immune responses. Ruminants such as sheep and cattle are considered among the “γδ T cell high” species, “γδ high” refering to the level of γδ T cells in circulation. Bovine γδ T cells were shown for example to respond to components of mycobacteria [5], to autologous antigens on monocytes [6]. The bovine is a valuable model to study respiratory disorders as coronaviruses [7] and influenza viruses [8]. Sheep is also a valuable model to study respiratory disorders as allergic asthma during pregnancy in relation with lung and immune development [9]. Several studies have already been done on the TRA/TRD locus of cattle [10,11,12,13] and sheep [14,15,16]. The complete genome assemblies, qualified as “representative genome”, are available at NCBI [17] for both species: ARS-UCD1.2 (de novo assembly, using long reads for assembly and short reads for scaffolding and polishing, of a Hereford cattle) for *Bos taurus*, and Oar_rambouillet_v1.0 (de novo assembly, using Pacific Biosciences, Menlo Park, CA, USA long reads for assembly, Illumina, San Diego, CA, USA short reads for polishing, and Hi-C Illumina data for scaffolding, of a Rambouillet sheep) for *Ovis aries*.

IMGT^®^, the international ImMunoGeneTics information system^®^, http://www.imgt.org [18], is the global reference in immunogenetics and immunoinformatics [1], founded in 1989 by Marie-Paule Lefranc at Montpellier (Université de Montpellier and CNRS). IMGT^®^ is a high-quality integrated knowledge resource specialized in the immunoglobulins (IG) or antibodies, T cell receptors (TR), major histocompatibility (MH) of human and other vertebrate species, and in the immunoglobulin superfamily (IgSF), the MH superfamily (MhSF) and related proteins of the immune system (RPI) of vertebrates and invertebrates.

IMGT has performed the biocuration of the TRA/TRD locus extracted from the representative genome ARS-UCD1.2 (*Bos taurus*) and Oar_rambouillet_v1.0 (*Ovis aries*) in order to provide a complete overview regarding the gene content and organization for both species. The aim of this study is to present the results of the annotation of *Bos taurus* and *Ovis aries* TRA/TRD loci and to highlight the differences of the newly annotated locus compared to the previous published genomic assemblies (UMD3.1 [13], Btau_3.1 [12] and Btau 4.0 [11] for *Bos taurus*; Oar_v3.1 for *Ovis aries* [16]). The comparison of the TRA/TRD locus for both species and human is also provided.

## 2. Materials and Methods

### 2.1. Annotation of the TRA/TRD Locus

The IMGT biocuration pipeline for locus annotation has been described previously [19]. Each locus sequence was localized on the corresponding chromosome and subsequently extracted from NCBI assembly [17] in GenBank format. The delimitation of the locus was performed by the search of the “IMGT bornes” which are coding genes (other than IG or TR) conserved between species, located upstream of the first or downstream of the last gene of an IG or TR locus (http://www.imgt.org/IMGTindex/IMGTborne.php). The IMGT 5′ borne of the TRA/TRD locus is the olfactory receptor 10G3 (OR10G3) gene and the IMGT 3′ borne of the locus is the defender against cell death (DAD1) gene. The locus orientation on a chromosome can be either forward (FWD) or reverse (REV) therefore the REV locus sequences were placed in the 5′ to 3′ locus orientation. Each locus sequence was assigned to an IMGT^®^ accession number (bovine: IMGT000049 (CM008177.2 (22253137-25584362, complement)) and sheep: IMGT000048 (CM008478.1 (23556113-26437716, complement))).

The biocuration has been performed manually assisted by internally developed tools (IMGT/LIGMotif [20], NtiToVald and IMGT/Automat [21]) based on the IMGT-ONTOLOGY axioms and concepts: ‘IDENTIFICATION’, ‘DESCRIPTION’, ‘CLASSIFICATION’, ‘NUMEROTATION’, ‘LOCALIZATION’, ‘ORIENTATION’ and ‘OBTENTION’ [22]. IMGT-ONTOLOGY includes the controlled vocabulary and annotation rules which are indispensable to ensure accuracy, consistency and coherence.

The IMGT nomenclature [23] of all TRAV and TRDV genes, ‘CLASSIFICATION’ axiom of IMGT-ONTOLOGY, was characterized according to the human TRAV/TRDV genes using IMGT/V-QUEST [24] and NGPhylogeny.fr [25] (using MAFFT [26] and PhyML [27] programs) to define the subgroups. TRAV/TRDV genes are designated by a number for the subgroup followed, whenever there are several genes belonging to the same subgroup, by a hyphen and a number picturing their relative localization in the locus. Numbers increase from 5′ to 3′ in the locus [3]. Two genes belong to the same subgroup if their identity percentage is greater than 75% in their V-REGION.

The IMGT unique numbering for the V-DOMAIN [28] and for the C-DOMAIN [29] (NUMEROTATION axiom) was used for the IMGT Colliers de Perles representations [30,31] and for the strands and loops delimitations of the V-REGION (FR1-IMGT to FR3-IMGT and CDR1-IMGT, CDR2-IMGT and germline CDR3-IMGT) and of the C-ALPHA and C-DELTA domains, respectively.

The functionality of the genes was defined according to the IMGT ‘functionality’ concept, part of the ‘IDENTIFICATION’ axiom of IMGT-ONTOLOGY, described in http://www.imgt.org/IMGTScientificChart/SequenceDescription/IMGTfunctionality.html. A gene is considered as functional if it has an open reading frame without stop codon, no defect in the splicing sites, recombination signals and/or regulatory elements; a gene is considered as ORF if the coding region has an open reading frame, but alterations in the splicing sites, recombination signals, regulatory elements and/or changes of conserved amino acids; a gene is considered as pseudogene if the coding region has stop codon(s) and/or frameshift mutation(s).

The main concept of the ‘DESCRIPTION’ axiom of IMGT-ONTOLOGY corresponds to IMGT^®^ standardized labels in the databases and tools. A set of specific labels was defined to describe the different organizations of IG and TR genes in clusters at the scale of the locus or of the chromosome. They are available from the IMGT/LIGM-DB database http://www.imgt.org/ligmdb/label#.

The standardized annotation allows data entry in the IMGT^®^ reference directory used in IMGT^®^ databases and tools (IMGT/LIGM-DB [32], IMGT/GENE-DB [33], IMGT/ 3Dstructure-DB and IMGT/2Dstructure-DB [34], IMGT/V-QUEST [24], IMGT/HighV-QUEST [35] and IMGT/DomainGapAlign [36]). IMGT^®^ genomic annotated data are then synthesized in IMGT Repertoire (http://www.imgt.org/IMGTrepertoire/) including several organized web pages (Locus representation, Locus description, Locus in genome assembly, Locus gene order, Gene tables, Potential germline repertoire, Protein displays, Alignments of alleles, Colliers de Perles [30,31], and [CDR1-IMGT.CDR2-IMGT.CDR3-IMGT] lengths) [19].

### 2.2. Comparison of the TRA/TRD Locus

The data obtained by biocuration were compared to the human TRA/TRD locus. The human TRA/TRD locus is located on chromosome 14 (14q11.2) on FWD orientation and spans 1000 kilobases (kb) [3]. The IMGT 5′ borne (OR10G3) has been identified 51 kb upstream of the first gene of the locus and the IMGT 3′ borne (DAD1), 13 kb downstream (in 3′) of the last gene of the locus. The potential repertoire consists of a total of 64 V genes: 56 TRAV genes (38 functional (F), 16 pseudogenes (P) and 2 F or P (depending on alleles)) belonging to 42 TRAV subgroups, 3 TRDV genes (F) belonging to 3 TRDV subgroups and 5 TRAV/DV genes (4 F and 1 F or P (depending on alleles)) belonging to 5 subgroups, 3 TRDD genes (F), 65 J genes: 61 TRAJ genes (50 F, 7 ORF, 3 P and 1 F or P) and 4 TRDJ genes (F), 1 TRAC gene (F) and 1 TRDC gene (F) [3].

A comparison was performed based on the number of genes in the locus as well as the number of genes per subgroup (potential germline repertoire), the locus representation, the functionality of genes and the CDR lengths. Potential duplications and/or deletions that may have occurred during evolution are susceptible to be highlighted from this sort of comparison.

## 3. Results

### 3.1. Annotation of TRA/TRD Loci

The two TRA/TRD loci were annotated following the pipeline described in the Materials and Methods. The results of the annotation described below are summarized in Table 1. The information regarding the genome assemblies and the boundaries is provided in Supplementary Table S1.

The bovine TRA/TRD locus, on chromosome 10 (REV), spans 3331 kb and consists of a total of 238 V genes: 183 TRAV genes (79 F, 14 ORF, 74 P, 3 F or ORF, 9 F or P, 3 ORF or P and 1 F or ORF or P) belonging to 40 TRAV subgroups and 39 (+16 non localized) TRDV genes (45 F, 5 ORF and 5 P) belonging to 5 TRDV subgroups, 9 TRDD genes (6 F and 3 ORF), 64 J genes: 60 TRAJ genes (52 F, 2 ORF, 4 P and 2 F or P) and 4 TRDJ genes (3 F and 1 ORF), 1 TRAC gene (F) and 1 TRDC gene (F). The IMGT 5’ borne (OR10G3) has been identified 24 kb upstream of the first gene of the locus and the IMGT 3’ borne (DAD1), has been identified 12 kb downstream of the last gene of the locus (cf. Supplementary Figure S1).

The sheep TRA/TRD locus, on chromosome 7 (REV), spans 2882 kb and consists of a total of 381 V genes: 277 (+16 non localized) TRAV genes (124 F, 11 ORF, 149 P, 1 F or ORF, 7 F or P and 1 ORF or P) belonging to 39 TRAV subgroups and 70 (+18 non localized) TRDV genes (34 F, 12 ORF, 28 P, 5 F or ORF, 6 F or P and 3 ORF or P) belonging to 5 TRDV subgroups, 9 TRDD genes (5 F and 4 ORF), 84 J genes: 79 (+1 non localized) TRAJ genes (61 F, 6 ORF and 13 P) and 4 TRDJ genes (3 F and 1 ORF), 1 TRAC gene (F) and 1 TRDC gene (F). The IMGT 5′ borne (OR10G3) was not found and IMGT 3′ borne (DAD1) has been identified 12 kb downstream of the last gene of the locus (cf. Supplementary Figure S2).

### 3.2. Comparison with Previous Studies

Regarding the sequences and the number of gaps, the quality of the last assemblies (this study) is better than the previous studies. For the bovine, the entire locus is localized on the chromosome 10 and there is only seven gaps. In all the previous assemblies there are genes on unplaced scaffolds and there are more than 260 gaps, except for [11]. On the other hand, many more genes have been described in previous studies (cf. Table 2). For the sheep, the entire locus is localized on the chromosome 7 and there are eighteen gaps. In the previous assembly there are genes on unplaced scaffolds and there are more than 80 gaps. Unlike cattle, fewer genes have been described in previous studies (cf. Table 3).

Given that there is access to two full assemblies (ARS-UCD1.2 for *Bos taurus* and Oar_rambouillet_v1.0 for *Ovis aries*), qualified as “representative genome” and as the corresponding TRA/TRD locus has been fully localized on a single chromosome with fewer gaps than in previous IMGT annotated genomic sequences, IMGT000049 and IMGT000048 are considered as IMGT references loci. It has allowed the establishment of the bovine and sheep TRA/TRD gene nomenclature, as well as the evaluation of the functionality of genes. The previous IMGT genomic sequences were re-annotated accordingly and the allelic variants determined based on nucleotide differences in the core region (V-REGION, D-REGION, J-REGION, C-REGION).

### 3.3. Comparison of the TRA J-C-CLUSTER

The number of TRAJ genes of human and bovine is similar and there are 19 more genes in sheep (cf. Table 1). Two TRAJ genes (TRAJ51 and TRAJ55) are missing in cattle and sheep compared to humans, and there are two TRAJ8 genes while there is only one in human. (cf. Table 4). The 19 TRAJ supplementary genes found in the sheep as a consequence of a duplication (or triplication for some genes) from TRAJ29 to TRAJ39 maybe due to a sequencing error or an amplification. Regarding the functionality, TRAC genes are functional and few TRAJ genes are P in human and bovine (3–4 and 4–6, depending on alleles, respectively). On the other hand, there are more pseudogenes in sheep mostly due to the duplicated genes (11 P out of 13 are duplicated genes) (cf. Table 4).

**Figure 1:**
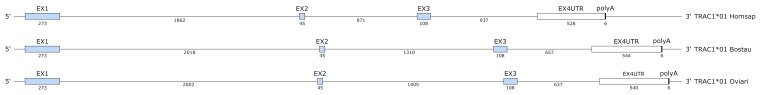
Structure of the TRAC genes in human (Homsap), bovine (Bostau) and sheep (Oviari). The numbers correspond to the size of the exons and introns in nucleotides.

**Figure 2:**
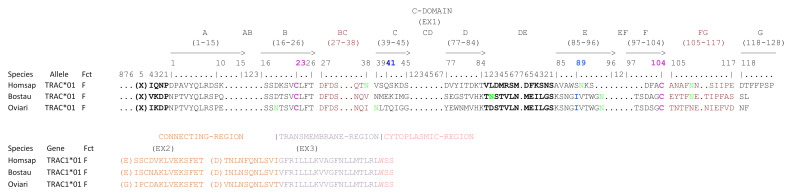
Protein display of the TRAC genes in human (Homsap), bovine (Bostau) and sheep (Oviari). Only alleles *01 are shown. The description of the strands and loops is according to the IMGT unique numbering for C-DOMAIN [29]. The AA between parentheses at the beginning of EX1, EX2 and EX3 corresponds to the first codon resulting from a splicing frame 1 (sf1). (http://www.imgt.org/IMGTeducation/Aide-memoire/_UK/splicing/). Data available in IMGT Repertoire (IG and TR) http://www.imgt.org/IMGTrepertoire/ > Proteins and alleles > Protein displays > C-DOMAIN > TRAC > Human, ibid. Bovine, ibid. Sheep.

At the genomic level, each TRAC gene consists of several exons whose sizes are the same for all species except for exon 4 which is untranslated (EX4UTR) (cf. Figure 1). On the other hand, the size of the introns varies according to the species, especially between human and bovine/sheep. In humans, the intron between the exon 1 (EX1) and the exon 2 (EX2) and the intron between EX2 and the exon 3 (EX3) are shorter while the intron between EX3 and EX4UTR is longer compared to bovine and sheep. Each TRAC gene encodes a similar protein of 142 AA with the exon 1 (EX1) encoding the constant domain, the exon 2 (EX2) and the 5’ part of the exon 3 (EX3) encoding the connecting region, the middle of EX3 encoding the transmembrane region and the 3’ part of EX3 encoding the cytoplasmic region (cf. Figure 2). Nevertheless, the structure of EX1 is different, there are fewer AA in the E and F strand and more AA in the G strand of human TRAC compared to bovine/sheep.

### 3.4. Comparison of the TRD D-J-C-CLUSTER

The number of TRDJ genes of human, bovine and sheep is the same but there are more TRDD genes in bovine and sheep (nine against three in human) (cf. Table 1). Regarding the functionality, TRDC genes are functional, few TRDD genes are ORF in bovine and sheep (three and four, respectively) (cf. Table 5) and one TRDJ gene is ORF both in bovine and sheep (TRDJ2) (cf. Table 6).

**Figure 3:**
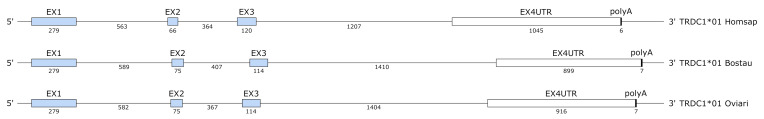
Structure of the TRDC genes in human (Homsap), bovine (Bostau) and sheep (Oviari). The numbers correspond to the size of the exons and introns in nucleotides.

**Figure 4:**
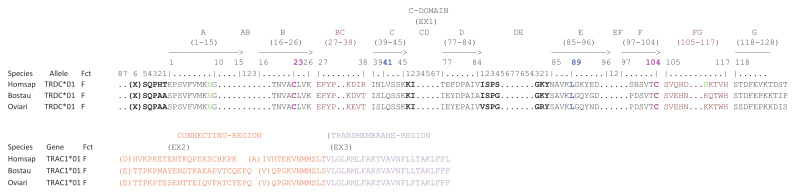
Protein display of the TRDC genes in human (Homsap), bovine (Bostau) and sheep (Oviari). Only alleles *01 are shown. The description of the strands and loops is according to the IMGT unique numbering for C-DOMAIN [29]. The AA between parentheses at the beginning of EX1, EX2 and EX3 corresponds to the first codon resulting from a splicing frame 1 (sf1). (http://www.imgt.org/IMGTeducation/Aide-memoire/_UK/splicing/). Data available in IMGT Repertoire (IG and TR) http://www.imgt.org/IMGTrepertoire/ > Proteins and alleles > Protein displays > C-DOMAIN > TRAC > Human, ibid. Bovine, ibid. Sheep.

Unlike TRAC, the size of the exons of TRDC varies depending on the species except for EX1 (cf. Figure 3). The EX2 is shorter in human but the EX3 is longer compared to bovine and sheep. In the same way, the size of the introns varies according to the species. Each TRDC gene encodes a similar protein of 155-156 AA with EX1 encoding the constant domain, EX2 and the 5′ part of EX3 encoding the connecting region and the 3′ part of EX3 encoding the transmembrane region (cf. Figure 4).

### 3.5. Comparison of the V-CLUSTER

**Figure 5:**
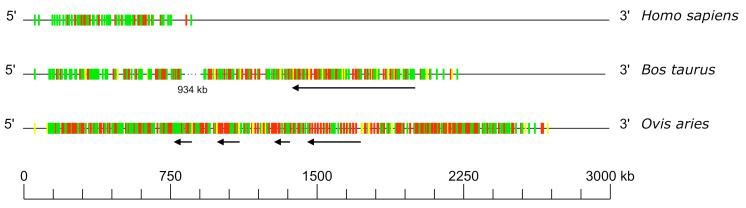
Schematic comparison of the TRA/TRD V-CLUSTER among human (*Homo sapiens*), bovine (*Bos taurus*) and sheep (*Ovis aries*). Colors are according to IMGT color menu for genes (http://www.imgt.org/IMGTScientificChart/RepresentationRules/colormenu.php#h1_28): in green: functional genes, in yellow: ORF genes and in red: pseudogenes. The dotted line in *Bos taurus* indicates the distance in kb between two genes not represented at scale. Data available in IMGT Repertoire (IG and TR) http://www.imgt.org/IMGTrepertoire/ > Locus and genes > Locus representations > TRA, ibid. TRD > Human, ibid. Bovine, ibid. Sheep.

The size of the V-CLUSTER (which describes the principal set of TRAV/TRDV genes) varies (cf. Figure 5). The V-CLUSTER is less extensive in human (56 genes on 900 kb) than in the bovine and sheep, which is consistent with the number of genes in these species (221 genes over 2200 kb and 346 genes on 2700 kb, respectively). Regarding the functionality of V genes, the proportion of functional genes is more important in human and in bovine compared to pseudogenes. However, there are more pseudogenes in sheep.

#### 3.5.1. Comparison of the TRAV genes

**Figure 6:**
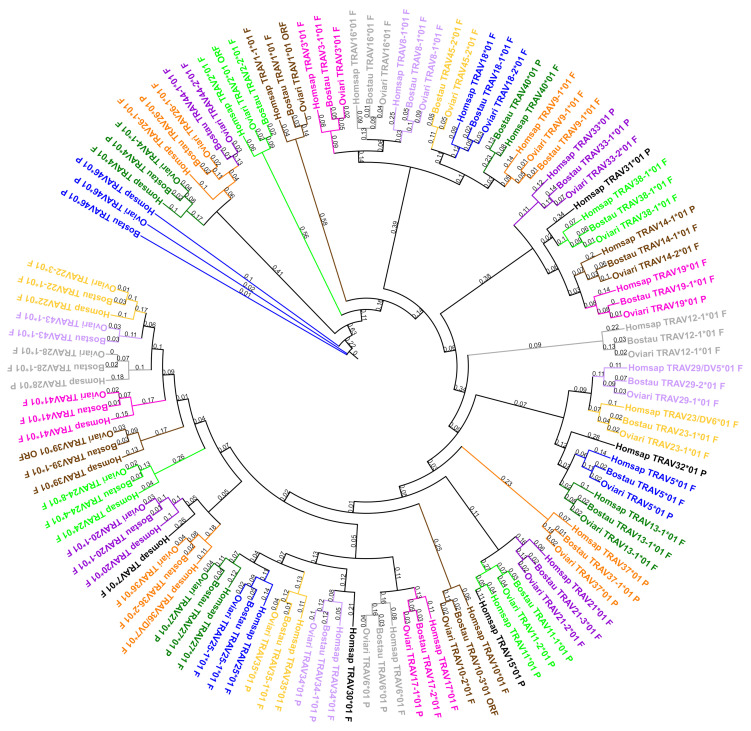
Phylogenetic tree of all TRAV subgroups for all species with one representative gene per subgroup (using V-REGION). Homsap: human, Bostau: bovine and Oviari: sheep. The different colors highlight the different subgroups. In black: subgroups only present in humans. Tree generated using NGPhylogeny.fr [25] (with MAFFT [26] and PhyML [27] programs) and iTOL v4 [37].

All subgroups were defined according to those of the human genome. A phylogenetic tree with one representative gene by subgroup (except for TRAVA, TRAVB and TRAVC, highly degenerated pseudogenes present only in human) for the human, the bovine and the sheep was created in order to highlight the distance between the species within a subgroup (cf. Figure 6). This phylogenetic tree shows that, for the two species, the genes of a subgroup are grouped in the same branch with a corresponding human gene. Nonetheless there are subgroups missing in both cattle and sheep (TRAV7, TRAV15, TRAV30, TRAV31, TRAV32, TRAVA, TRAVB and TRAVC) and only in sheep (TRAV40), new subgroups in bovine and sheep (TRAV43, TRAV44 and TRAV45) and three subgroups are intermingled: TRAV4, TRAV26 and TRAV44 (cf. Supplementary Figure S3). However, there is less than 75% identity among the genes of these three subgroups for a given species, so they cannot be considered as genes belonging to the same subgroup.

The number of TRAV genes varies depending on the species. There are fewer genes in human than in bovine and fewer genes in bovine than in sheep (cf. Table 1). The number of genes per subgroup also varies according to the species (cf. Table 7). In humans there are one or two genes by subgroup except for TRAV8 and TRAV12 (eight and three genes, respectively) while in cattle and sheep there are subgroups highly developed. In the sheep, there are six subgroups with more than 20 genes (TRAV8, TRAV13, TRAV22, TRAV23, TRAV25 and TRAV44) and three subgroups with more than 10 genes (TRAV9, TRAV14 and TRAV43) although there are only five subgroups in bovine with more than 10 genes (TRAV22, TRAV23, TRAV25, TRAV44 and TRAV45). In addition, as show in the phylogenetic tree (cf. Figure 6) eight subgroups are absent in both species and one subgroup is missing only in sheep.

The CDR lengths are relatively well conserved between the different species (cf. Table 8). The most important differences are in bovine where for some subgroups there are two or three different lengths (TRAV10, TRAV20, TRAV22 and TRAV38) and for three human subgroups in which the CDR length is different from bovine and sheep (TRAV11, TRAV35 and TRAV39). These differences are shown in red in Table 8. For two subgroups (TRAV17 and TRAV18) the bovine has some genes with the same CDR lengths as human (in blue) and some with the same CDR lengths as sheep (in green).

#### 3.5.2. Comparison of the TRDV genes

**Figure 7:**
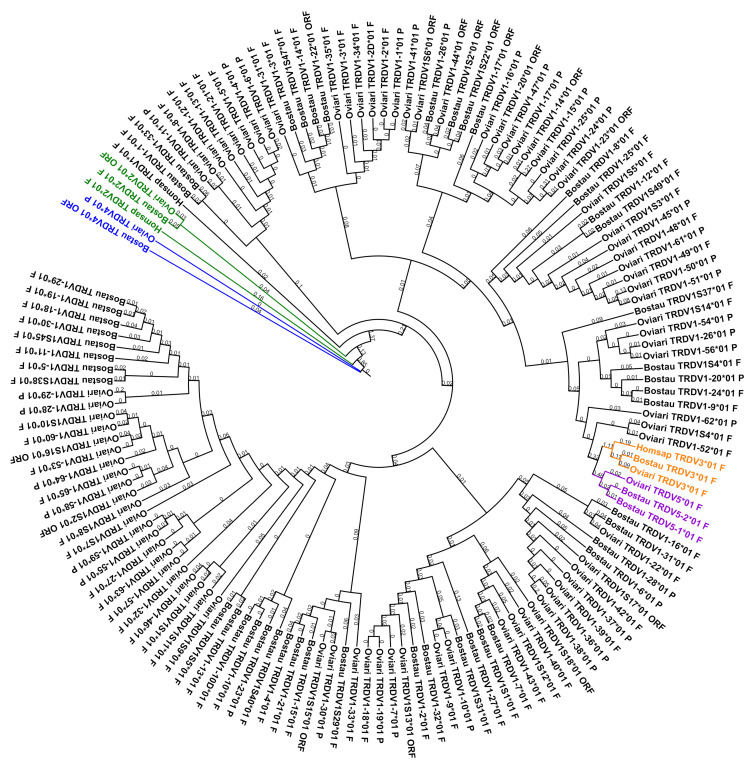
Phylogenetic tree of all TRDV genes for all species (using V-REGION). Homsap: human, Bostau: bovine and Oviari: sheep. The different colors highlight the different subgroups. Tree generated using NGPhylogeny.fr [25] (with MAFFT [26] and PhyML [27] programs) and iTOL v4 [37].

Like for the TRAV genes, the subgroups were defined according to those of the human genome and a phylogenetic tree with all genes was created (cf. Figure 7). This phylogenetic tree shows that, except for the TRDV1 subgroup, the genes are grouped in the same branch with a corresponding human gene. However the TRDV1 subgroup is divided in two branches even if there is more than 75% identity between all those genes.

As for the TRAV genes, the number of TRDV genes varies depending on the species. There are fewer genes in human than in bovine and fewer genes in bovine than in sheep (cf. Table 1). There are two new subgroups in bovine and sheep compared to human (TRDV4 and TRDV5) and the TRDV1 subgroup much larger in cattle and sheep with 50 and 84 genes, respectively, compared to 1 in human (cf. Table 9).

**Figure 8:**
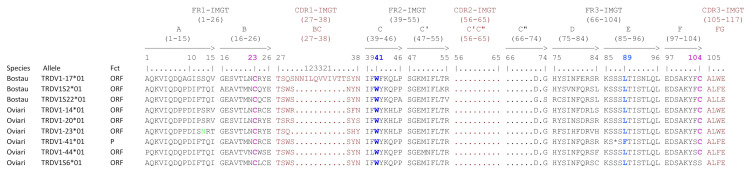
Protein display of the TRDV1 genes with lack of CDR2-IMGT and part of FR3-IMGT in bovine (Bostau) and sheep (Oviari). Only alleles *01 are shown. The description of the strands and loops is according to the IMGT unique numbering for V-REGION [28]. Data available in IMGT Repertoire (IG and TR) http://www.imgt.org/IMGTrepertoire/ > Proteins and alleles > Protein displays > V-REGION > TRDV > Human, ibid. Bovine, ibid. Sheep.

Contrary to TRAV genes, the CDR lengths are not conserved between human and bovine/sheep for TRDV2 and TRDV3 subgroups (cf. Table 10). For TRDV1 subgroups, there are several different lengths for bovine and sheep (nine and five respectively) due to the high number of genes in this subgroup. There are also genes with lack of CDR2-IMGT and part of CDR3-IMGT (deletion of nine amino acids (AA), not shown in Table 10). This particularity was already described in bovine by [11] and is present in sheep too. Four genes are concerned in bovine (three in-frame and one out-of-frame (P with frameshift)) and two in sheep (six in-frame and two out-of-frame). The in-frame genes are shown in Figure 8.

### 3.6. Analysis of the cDNA Sequences

The last step of the biocuration pipeline consists of the automatic annotation of the cDNAs available in IMGT/LIGM-DB database with the IMGT/Automat tool [21]: 176 cDNA sequences for cattle and 102 for sheep were annotated. This annotation allowed to highlight the transcription of approximately 50% (for cattle) and 40% (sheep) of the germline genes. Interestingly, TRAJ54 which has a stop codon in position 1 of the J-REGION, and TRDV1-13 with a stop codon in position 108, last position of the V-REGION have been found rearranged and give a productive sequence (with no stop codon and an in-frame junction) in accessions numbers JX065661 (http://www.imgt.org/ligmdb/result.action?accessionNumber=JX065661) and BC113229 (http://www.imgt.org/ligmdb/result.action?accessionNumber=BC113229) respectively, showing the trimming of the stop codon during the rearrangement.

## 4. Discussion

This study was carried out in order to highlight the differences between the IMGT^®^ annotation and the data previously published and to compare the TRA/TRD loci among bovine and sheep against the human locus. The annotation of each locus followed the pipeline defined in Materials and Methods. The expertise that follows this pipeline permits to establish the TRA/TRD germline repertoire according to IMGT^®^ nomenclature and the IMGT^®^ reference directory (IMGT^®^ reference sequences used by IMGT^®^ tools) of each locus and thus obtain sequence, gene and structure data. For each gene analyzed, there are more than 200 pieces of information available in IMGT^®^ databases, tools and web pages. The comparison of the data obtained after the biocuration was carried out against the data of the human TRA/TRD loci. This analysis was done with respect to the data entered in IMGT Repertoire.

The two loci in the last assemblies have fewer gaps and are localized on a chromosome without unplaced scaffold compared to the previous studies (cf. Table 2 and Table 3). Indeed, it is a basic requirement, with an expected positional organization of genes in the locus, for the annotation of a complete locus with a definitive nomenclature in IMGT^®^. We rely on publicly available data, which is why we need good quality data so that we can annotate what we see with good quality annotations.

It is worth noting that the nomenclature presented in this manuscript, for the under question loci and species, is carved on stone and it will not change in the future. As a matter of fact, once the IMGT biocuration team gets hold of a genomic assembly covering the whole locus (no contigs, no scaffolds), then a reference assembly is established which gives rise to the definite IMGT nomenclature. Obviously enough, subsequent assemblies might/will be available either for the same individual or for other individuals which will constitute novel haplotypes in the latter case, but will not afffect the original nomenclature.

During the analysis of the TRA/TRD locus in bovine and sheep, it was noted that the general organization of the locus is conserved and is similar to the human one even if the V-CLUSTER is more extensive (cf. Figure 5). It should be noted that the IMGT^®^ unique nomenclature, based on subgroup assignment and position of genes within the locus, represents a valuable help in highlighting locus organizational similarities or differences.

The results show that some subgroups are missing and three new subgroups were described in bovine and sheep compared to human. Some subgroups are more represented in bovine and in sheep than in human, which may indicate potential duplications during evolution. It can also explain the difference in the proportion of functional genes. Indeed, duplicated subgroups in bovine and sheep are composed of an important proportionality of pseudogenes resulting higher number of pseudogenes compared to human. Another indication of duplication during evolution is the presence of an important number of TRDV1 genes (50 in bovine and 66 in sheep) compared to 1 in human [13].

In the TRAV genes, there is only one CDR length for most of human, bovine and sheep subgroups, except for six bovine subgroups (TRAV10, TRAV17, TRAV18, TRAV20, TRAV22 and TRAV38) (cf. Table 8) while in the TRDV1 subgroups there are several lengths (cf. Table 10) and even some genes without CDR2-IMGT (cf. Figure 8).

It would be interesting to see if these specificities (expansion of the TRDV1 subgroup and of the TRAV subgroups, absence of CDR2-IMGT for some TRDV1 genes, etc.) are also found in other ruminant species.

The veterinary species are valuable models for immunological and medical research. The comparison of the TRA/TRD locus among bovine and sheep presented here allow to have a global vision of the TRA/TRD locus in Bovidae and will be a useful resource to analyze the TRA/TRD locus in new species not yet analyzed. The work carried out and the use of the methodology established for the analysis of the TRB locus [19] show that this procedure can be used to facilitate the analysis of IG (IGH, IGK and IGL) and TR (TRA, TRB, TRD and TRG) loci among different species.

## Figures and Tables

**Table 1:** Results of the analysis of TRA/TRD loci in human (*Homo sapiens*), bovine (*Bos taurus*) and sheep (*Ovis aries*).

Species	*Homo sapiens*	*Bos taurus*	*Ovis aries*
Chromosome (Orientation)	14 (forward (FWD))	10 (REV)	7 (REV)
Size (kb)	1000	3331	2882
Number of TRAV genes	56	183	277 (+16 non localized)
Number of TRDV genes	3	39 (+ 16 non localized)	70 (+18 non localized)
Number of TRAV/DV genes	5	0	0
Number of TRDD genes	3	9	9
Number of TRAJ genes	61	60	79 (+1 non localized)
Number of TRDJ genes	4	4	4
Number of TRAC genes	1	1	1
Number of TRDC genes	1	1	1

Data available in IMGT Repertoire (IG and TR) http://www.imgt.org/IMGTrepertoire/ > Locus and genes > Locus descriptions > Locus description > TRA, ibid. TRD > Human, ibid. Bovine, ibid. Sheep.

**Table 2:** Comparison of the different studies of TRA/TRD loci in bovine (*Bos taurus*).

	This Study	Connelley et al., 2014 [13]	Herzig et al., 2010, [12]	Reinink and Van Rhijn, 2009 [11]
Assembly	ARS-UCD1.2	UMD3.1	Btau_3.1	Btau4.0
Chromosome	chr 10	chr 10 + chr 9 + 1 unplaced scaffold	2 scaffolds on chr 10 + 27 unplaced scaffold	chr 10 + 3 unplaced scaffolds
Number of gap	7	Around 260	265	5
Number of TRAV	183	306	NA	318
Number of TRDV	39	65	56	80
Number of TRDD	9	5	5	NA
Number of TRAJ	60	62	NA	NA
Number of TRDJ	4	3	3	NA
Number of TRAC	1	1	NA	NA
Number of TRDC	1	1	1	NA

chr: chromosome; NA: not applicable.

**Table 3:** Comparison of the different studies of TRA/TRD loci in sheep (*Ovis aries*).

	This Study	Piccinni et al., 2015 [16]	Antonacci et al., 2005 [15]
Assembly	Oar_rambouillet_v1.0	Oar_v3.1	individual sequences
Chromosome	chr 7	chr 7 + 25 unplaced scaffolds	NA
Number of gap	18	83	NA
Number of TRAV	277	66	NA
Number of TRDV	70	25	23
Number of TRDD	9	7	NA
Number of TRAJ	79	61	NA
Number of TRDJ	4	4	NA
Number of TRAC	1	1	NA
Number of TRDC	1	1	NA

chr: chromosome; NA: not applicable.

**Table 4:** IMGT Potential germline repertoires of the TRAJ sets in human (*Homo sapiens*), bovine (*Bos taurus*) and sheep (*Ovis aries*).

Sets	*Homo sapiens*	*Bos taurus*	*Ovis aries*
TRAJ1	1 O	1 F	1 F
TRAJ2	1 O	1 F	1 F
TRAJ3	1 F	1 F	1 O (2)
TRAJ4	1 F	1 FP (2)	1 O
TRAJ5	1 F	1 F	1 F (2)
TRAJ6	1 F	1 F	1 O
TRAJ7	1 F	1 F	1 F
TRAJ8	1 FP (2)	2 F (3)	2 F
TRAJ9	1 F	1 F	1 F (2)
TRAJ10	1 F	1 F	1 F (2)
TRAJ11	1 F	1 F (2)	1 F
TRAJ12	1 F	1 F	1 F
TRAJ13	1 F (2)	1 P (2)	1 O
TRAJ14	1 F	1 P (2)	1 O
TRAJ15	1 F (2)	1 FP (2)	1 F
TRAJ16	1 F (2)	1 F	1 F
TRAJ17	1 F	1 F	1 F
TRAJ18	1 F	1 F	1 F
TRAJ19	1 O	1 P	1 P
TRAJ20	1 F	1 F	1 F
TRAJ21	1 F	1 F	1 F
TRAJ22	1 F	1 F	1 F
TRAJ23	1 F (2)	1 F	1 F
TRAJ24	1 F (3)	1 F	1 F
TRAJ25	1 O	1 F (2)	1 F
TRAJ26	1 F	1 O	1 F
TRAJ27	1 F	1 F	1 F
TRAJ28	1 F	1 F	1 F
TRAJ29	1 F	1 F	2 F, 1 O
TRAJ30	1 F	1 F	1 F, 1 P
TRAJ31	1 F	1 F (2)	1 F, 2 P
TRAJ32	1 F (2)	1 F	2 F (3), 1 P
TRAJ33	1 F	1 F	2 F, 1 P
TRAJ34	1 F	1 F	2 F, 1 P
TRAJ35	1 F	1 O	2 F (3), 1 P
TRAJ36	1 F	1 F	2 F, 1 P
TRAJ37	1 F (2)	1 F (2)	2 F (3), 1 P
TRAJ38	1 F	1 F	1 F, 1 P
TRAJ39	1 F	1 F (2)	1 F, 1 P
TRAJ40	1 F	1 F	1 F
TRAJ41	1 F	1 F (2)	1 F
TRAJ42	1 F	1 F	1 F
TRAJ43	1 F	1 F	1 F
TRAJ44	1 F	1 F	2 F
TRAJ45	1 F	1 F (2)	1 F
TRAJ46	1 F	1 F	1 F
TRAJ47	1 F (2)	1 F	1 F
TRAJ48	1 F	1 F	1 F
TRAJ49	1 F	1 F	1 F
TRAJ50	1 F	1 F	1 F
TRAJ51	1 P	-	-
TRAJ52	1 F	1 F	1 F
TRAJ53	1 F	1 F	1 F
TRAJ54	1 F	1 F	1 F
TRAJ55	1 P	-	-
TRAJ56	1 F	1 F	1 F
TRAJ57	1 F	1 F	1 F
TRAJ58	1 O	1 F	1 F
TRAJ59	1 O	1 F	1 F
TRAJ60	1 P	1 F	1 F
TRAJ61	1 O	1 P	1 P
Total per Fct	50 F + 7 O + 3 P + 1 FP	52 F + 2 O + 4 P + 2 FP	61 F + 6 O + 13 P
Total genes	61 (71)	60 (72)	80 (87)

For each TRAJ set, in each species, the number of TRAJ genes by functionality and, between parentheses, the number of alleles are shown. F: functional; O: ORF; P: pseudogene; FP: genes with alleles of different functionalities. Data available in IMGT Repertoire (IG and TR) http://www.imgt.org/IMGTrepertoire/ > Locus and genes > Potential germline repertoires > TRAV and TRAJ > Human, ibid. Bovine, ibid. Sheep.

**Table 5:** IMGT Potential germline repertoires of the TRDD sets in human (*Homo sapiens*), bovine (*Bos taurus*) and sheep (*Ovis aries*).

Sets	*Homo sapiens*	*Bos taurus*	*Ovis aries*
TRDD1	1 F	1 O	1 O (2)
TRDD2	1 F	1 F	1 F
TRDD3	1 F	1 O	1 O
TRDD4	-	1 F	1 F
TRDD5	-	1 O	1 O
TRDD6	-	1 F	1 F
TRDD7	-	1 F	1 O
TRDD8	-	1 F	1 F
TRDD9	-	1 F	1 F
Total per Fct	3 F	6 F + 3 O	5 F + 4 O
Total genes	3 (3)	9 (9)	9 (10)

For each TRDD set, in each species, the number of TRDD genes by functionality and, between parentheses, the number of alleles are shown. F: functional; O: ORF. Data available in IMGT Repertoire (IG and TR) http://www.imgt.org/IMGTrepertoire/ > Locus and genes > Potential germline repertoires > TRDV, TRDD and TRDJ > Human, ibid. Bovine, ibid. Sheep.

**Table 6:** IMGT Potential germline repertoires of the TRDJ sets in human (*Homo sapiens*), bovine (*Bos taurus*) and sheep (*Ovis aries*).

Sets	*Homo sapiens*	*Bos taurus*	*Ovis aries*
TRDJ1	1 F	1 F	1 F
TRDJ2	1 F	1 O	1 O
TRDJ3	1 F	1 F	1 F
TRDJ4	1 F	1 F	1 F
Total per Fct	4 F	3 F + 1 O	3 F + 1 O
Total genes	4 (4)	4 (4)	4 (4)

For each TRDJ set, in each species, the number of TRDJ genes by functionality and, between parentheses, the number of alleles are shown. F: functional; O: ORF. Data available in IMGT Repertoire (IG and TR) http://www.imgt.org/IMGTrepertoire/ > Locus and genes > Potential germline repertoires > TRDV, TRDD and TRDJ > Human, ibid. Bovine, ibid. Sheep.

**Table 7:** IMGT Potential germline repertoires of the TRAV subgroups in human (*Homo sapiens*), bovine (*Bos taurus*) and sheep (*Ovis aries*).

Subgroups	*Homo sapiens*	*Bos taurus*	*Ovis aries*
TRAV1	2 F (5)	1 F	1 O
TRAV2	1 F (2)	5 F, 2 P	1 O (2)
TRAV3	1 FP (2)	6 F , 1 FP (2)	1 F (2)
TRAV4	1 F	1 P	2 F (3)
TRAV5	1 F	1 F (3)	1 FP (2)
TRAV6	1 F (7)	1 P (2)	1 P (2)
TRAV7	1 F	-	-
TRAV8	5 F (17), 3 P (4)	1 F (3), 4 P (6)	5 F (6), 22 P (24), 1 FO (2), 2 FP (4)
TRAV9	2 F (5)	4 F (7), 2 P	7 F (8), 5 P (7)
TRAV10	1 F (2)	1 O, 2 P	1 F (2), 1 P
TRAV11	2 P	3 P	3 P (4)
TRAV12	3 F (7)	2 F, 1 FP (3)	5 F (6), 2 P
TRAV13	2 F (5)	2 F (4), 2 P (4)	11 F, 10 P
TRAV14	1 F (4), 1 P (2)	1 F, 1 O (2), 1 P, 1 FP (3)	7 F, 1 O, 6 P
TRAV15	1 P	-	-
TRAV16	1 F	1 F	1 F (2)
TRAV17	1 F	1 F (2), 2 P	1 P, 1 FP (2)
TRAV18	1 F	1 F, 2 O, 1 P (2), 1 FO (3)	3 F, 1 P
TRAV19	1 F	2 F, 1 P (2), 1 FOP (3)	1 P
TRAV20	1 F (4)	2 F (3), 1 O, 1 P	2 F, 2 P
TRAV21	1 F (2)	2 F (4), 1 O, 1 P	2 F (3), 3 P
TRAV22	1 F	6 F (7), 3 O, 5 P (7), 1 FO (3), 1 FP (3)	20 F (22), 22 P (24), 1 FP (2)
TRAV23	1 F (5)	1 F (2), 10 P (14), 2 OP (4)	5 F, 2 O, 32 P (37)
TRAV24	1 F (2)	1 F , 5 P (7), 1 FO (2)	1 F, 2 O, 5 P
TRAV25	1 F	4 F (6), 1 O, 9 P, 1 FP (2)	10 F (11), 2 O, 13 P, 1 FP (2)
TRAV26	2 F (5)	1 P (3)	2 F (3)
TRAV27	1 F (3)	1 OP (2)	1 FP (2)
TRAV28	1 P (2)	2 F (4), 1 P	2 F (4)
TRAV29	1 FP (4)	1 F (3), 1 O	2 F (3)
TRAV30	1 F (5)	-	-
TRAV31	1 P (2)	-	-
TRAV32	1 P	-	-
TRAV33	1 P	2 P (3), 1 FP (4)	1 F, 1 P (2)
TRAV34	1 F	2 P (4)	1 P
TRAV35	1 FP (3)	2 F	1 P (2)
TRAV36	1 F (5)	1 F, 1 P	1 F (2)
TRAV37	1 P	2 P	1 P
TRAV38	2 F (5)	6 F (9)	2 F (3)
TRAV39	1 F	1 F, 1 O	1 O (2)
TRAV40	1 F	1 P	-
TRAV41	1 F	1 F	1 F (2)
TRAV43	-	3 F (6)	13 F (15), 2 P (3)
TRAV44	-	8 F, 1 O, 8 P (11), 1 FP (2)	13 F (14), 1 O, 11 P (12)
TRAV45	-	10 F (14), 1 O, 2 P (3), 2 FP (4)	4 F (5), 1 P, 1 OP (2)
TRAV46	1 P	1 P	1 P (2)
TRAVA	1 P (2)	-	-
TRAVB	1 P (2)	-	-
TRAVC	1 P	-	-
Total per Fct	42 F + 16 P + 3 FP	79 F + 14 O + 74 P + 3 FO + 9 FP + 3 OP + 1 FOP	124 F + 11 O + 149 P + 1 FO + 7 FP + 1 OP
Total genes	61 (134)	183 (263)	293 (344)

For each TRAV subgroup, in each species, the number of TRAV genes by functionality and, between parentheses, the number of alleles are shown. F: functional; O: ORF; P: pseudogene; FO, FP, PO, FOP: genes with alleles of different functionalities. Data available in IMGT Repertoire (IG and TR) http://www.imgt.org/IMGTrepertoire/ > Locus and genes > Potential germline repertoires > TRAV and TRAJ > Human, ibid. Bovine, ibid. Sheep.

**Table 8:** TRAV [CDR1-IMGT.CDR2-IMGT.CDR3-IMGT] lengths by subgroup and species in human (*Homo sapiens*), bovine (*Bos taurus*) and sheep (*Ovis aries*).

Subgroups	*Homo sapiens*	*Bos taurus*	*Ovis aries*
TRAV1	[6.6.3]	[6.6.3]	[6.6.3]
TRAV2	[6.4.3]	[6.4.3]	[6.4.3]
TRAV3	[6.8.4]	[6.8.3]	[6.8.3]
TRAV4	[7.5.4]	[7.5.4]	[7.5.4]
TRAV5	[6.7.3]	[6.7.3]	[6.7.3]
TRAV6	[6.7.3]	-	-
TRAV7	[6.7.3]	-	-
TRAV8	[6.8.3]	[6.8.3]	[6.8.3]
TRAV9	[6.7.3]	[6.7.3]	[6.7.3]
TRAV10	[6.7.3]	[5.7.3] [6.7.3]	[6.7.3]
TRAV11	[6.7.2]	[6.7.3]	[6.7.3]
TRAV12	[6.6.3]	[6.6.3]	[6.6.3]
TRAV13	[6.7.3]	[6.7.3]	[6.7.3]
TRAV14	[7.8.4]	[7.8.4]	[7.8.4]
TRAV16	[6.4.3]	[6.4.3]	[6.4.3]
TRAV17	[5.7.3]	[5.7.3] [6.7.3]	[6.7.3]
TRAV18	[6.6.3]	[6.6.3] [6.7.3]	[6.7.3]
TRAV19	[7.8.4]	[7.8.4]	[7.8.4]
		[4.7.3]	
TRAV20	[6.7.3]	[6.7.3]	[6.7.3]
TRAV21	[6.7.3]	[6.7.3]	[6.7.3]
		[4.5.3]	
TRAV22	[5.5.3]	[5.5.3]	[5.5.3]
TRAV23	[6.7.3]	[6.7.3]	[6.7.3]
TRAV24	[6.7.2]	[6.7.2]	[6.7.2]
TRAV25	[5.7.2]	[5.7.2]	[5.7.2]
TRAV26	[7.5.4]	[7.5.4]	[7.5.4]
TRAV27	[5.7.2]	[5.7.2]	
TRAV28		[5.5.3]	[5.5.3]
TRAV29	[6.7.3]	[6.7.3]	[6.7.3]
TRAV30	[5.7.3]	-	-
TRAV33	-	-	[7.7.5]
TRAV34	[5.7.3]	-	-
TRAV35	[5.7.3]	[5.7.2]	[5.7.2]
TRAV36	[6.7.3]	[6.7.3]	[6.7.3]
		[6.8.4]	
			
TRAV38	[7.8.4]	[7.8.4]	[7.8.4]
		[8.8.4]	
TRAV39	[5.7.3]	[6.7.3]	[6.7.3]
TRAV40	[6.4.3]	-	-
TRAV41	[5.5.3]	[5.5.3]	[5.5.3]
TRAV43	-	[5.5.3]	[5.5.3]
TRAV44	-	[7.5.4]	[7.5.4]
TRAV45	-	[7.7.3]	[7.7.3]

Only in-frame genes are considered. The differences in CDR length are shown in red. The correspondances for subgrousp TRAV17 and TRAV18 are shown in blue and green. Data available in IMGT Repertoire (IG and TR) http://www.imgt.org/IMGTrepertoire/ > 2D and 3D structures > FR-IMGT and CDR-IMGT lengths (V-REGION and V-DOMAIN) > [CDR1-IMGT.CDR2-IMGT.] length per subgroup > TRAV > Human, ibid. Bovine, ibid. Sheep.

**Table 9:** IMGT Potential germline repertoires of the TRDV subgroups in human (*Homo sapiens*), bovine (*Bos taurus*) and sheep (*Ovis aries*).

Subgroups	*Homo sapiens*	*Bos taurus*	*Ovis aries*
TRDV1	1 F	41 F (55), 4 O (5), 5 P	32 F (41), 11 O, 27 P (28), 5 FO (11), 6 FP (13), 3 OP (6)
TRDV2	1 F (3)	1 F	1 O (2)
TRDV3	1 F (2)	1 F	1 F (2)
TRDV4	-	1 O	1 P
TRDV5	-	2 F (4)	1 F (2)
Total per Fct	3 F	45 F + 5 O + 5 P	34 F + 12 O + 28 P + 5 FO + 6 FP + 3 OP
Total genes	3 (6)	55 (72)	88 (117)

For each TRDV subgroup, in each species, the number of TRDV genes by functionality and, between parentheses, the number of alleles are shown. F: functional; O: ORF; P: pseudogene; FO, FP, PO: genes with alleles of different functionalities. Data available in IMGT Repertoire (IG and TR) http://www.imgt.org/IMGTrepertoire/ > Locus and genes > Potential germline repertoires > TRDV, TRDD and TRDJ > Human, ibid. Bovine, ibid. Sheep.

**Table 10:** TRDV [CDR1-IMGT.CDR2-IMGT.CDR3-IMGT] lengths by subgroup and species in human (*Homo sapiens*), bovine (*Bos taurus*) and sheep (*Ovis aries*).

Subgroups	*Homo sapiens*	*Bos taurus*	*Ovis aries*
		[5.3.4]	
		[7.3.4]	
		[8.1.4]	[7.3.3]
		[8.3.4]	[7.3.4]
TRDV1	[7.3.4]	[9.3.3]	[7.3.5]
		[9.3.4]	[9.3.4]
		[9.3.15]	[13.3.4]
		[10.3.4]	
		[18.3.4]	
TRDV2	[8.3.4]	[9.3.4]	[9.3.4]
TRDV3	[7.6.2]	[7.6.4]	[7.6.4]
TRDV4	-	[8.3.4]	-
TRDV5	-	[7.3.5]	[7.3.5]

Only in-frame genes are considered. The differences in CDR length are shown in red. The correspondances for subgrousp TRDV1 are shown in blue. Data available in IMGT Repertoire (IG and TR) http://www.imgt.org/IMGTrepertoire/ > 2D and 3D structures > FR-IMGT and CDR-IMGT lengths (V-REGION and V-DOMAIN) > [CDR1-IMGT.CDR2-IMGT.] length per subgroup > TRDV > Human, ibid. Bovine, ibid. Sheep.

## Data Availability

The IMGT^®^ software and data are provided to the academic users and NPO’s (Not for Profit Organization(s)) under the CC BY-NC-ND 4.0 license. Any other use of IMGT^®^ material, from the private sector, needs a financial arrangement with CNRS.

## Supplementary Materials

The following are available online at https://www.mdpi.com/2073-4425/12/1/30/s1. Table S1: Information regarding the genome assembly and TRA/TRD locus IMGT 5′ and 3′ borne in human (*Homo sapiens*), bovine (*Bos taurus*) and sheep (*Ovis aries*). Figure S1: Locus representation of the bovine (*Bos taurus*) TRA/TRD locus. Colors are according to IMGT color menu for genes (http://www.imgt.org/IMGTScientificChart/RepresentationRules/colormenu.php#h1_28). The dotted line indicates the distance in kb between two genes not represented at scale. Figure S2: Locus representation of the sheep (*Ovis aries*) TRA/TRD locus. Colors are according to IMGT color menu for genes (http://www.imgt.org/IMGTScientificChart/RepresentationRules/colormenu.php#h1_28). Figure S3: Phylogenetic tree of all TRAV genes for all species (using V-REGION). Homsap: human, Bostau: bovine and Oviari: sheep. Tree generated using NGPhylogeny.fr [25] (with MAFFT [26] and PhyML [27] programs) and iTOL v4 [37].

## Abbreviations

The following abbreviations are used in this manuscript:

IG	Immunoglobulin
TR	T cell receptor
MH	Major histocompatibility
IgSF	Immunoglobulin Superfamily
MhSF	MH Superfamily
RPI	Related Protein of the Immune system
V	Variable
D	Diversity
J	Joining
C	Constant
OR10G3	Olfactory Receptor 10G3
DAD1	Defender Against cell Death
FWD	Forward
REV	Reverse
kb	kilobase
F	Functional
P	Pseudogene
AA	Amino acid
EX4UTR	Exon 4 untranslated
EX1	Exon 1
EX2	Exon 2
EX3	Exon 3
