# Supplementary material for: IMGT® Biocuration and Comparative Analysis of Bos taurus and Ovis aries TRA/TRD Loci
Source: Genes (Basel). 2020 Dec 28;12(1):30. doi: 10.3390/genes12010030 (PMC7824213; doi:10.3390/genes12010030)
Supplement: Supplementary file 1 [file genes-12-00030-s001.zip › References.docx]

References

Added references:

- 2. Lefranc, M.P.; Lefranc, G. The Immunoglobulin FactsBook; Academic Press, 2001.
- 5. Welsh, M.D.; Kennedy, H.E.; Smyth, A.J.; Girvin, R.M.; Andersen, P.; Pollock, J.M. Responses of bovine WC1(+) gammadelta T cells to protein and nonprotein antigens of Mycobacterium bovis. Infection and Immunity 2002, 70, 6114–6120. doi:10.1128/iai.70.11.6114-6120.2002.
- 6. Sathiyaseelan, T.; Naiman, B.; Welte, S.; Machugh, N.; Black, S.J.; Baldwin, C.L. Immunological characterization of a gammadelta T-cell stimulatory ligand on autologous monocytes. Immunology 2002, 105, 181–189. doi:10.1046/j.0019-2805.2001.01356.x.

Suppressed references:

- 17. Folch, G.; Jabado-Michaloud, J.; Bellahcene, F.; Regnier, L.; Giudicelli, V.; Lefranc, M.P. IMGT/Automat: the strategy for the annotation of human and mouse cDNA nucleotide sequences of IG and TR. Nature Precedings 2009. doi:10.1038/npre.2009.3159.1.
- 25. Lefranc, M.P. Unique database numbering system for immunogenetic analysis. Immunology Today 1997, 18, 509. doi:10.1016/s0167-5699(97)01163-8.
- 28. Ruiz, M.; Lefranc, M.P. IMGT gene identification and Colliers de Perles of human immunoglobulins with known 3D structures. Immunogenetics 2002, 53, 857–883. doi:10.1007/s00251-001-0408-6.
- 29. Kaas, Q.; Ehrenmann, F.; Lefranc,M.P. IG, TR and IgSF,MHC andMhcSF: what do we learn from the IMGT Colliers de Perles? Briefings in Functional Genomics & Proteomics 2007, 6, 253–264. doi:10.1093/bfgp/elm032.
- 35. Ehrenmann, F.; Lefranc, M.P. IMGT/3Dstructure-DB: querying the IMGT database for 3D structures in immunology and immunoinformatics (IG or antibodies, TR, MH, RPI, and FPIA). Cold Spring Harbor Protocols 2011, 2011, 750–761. doi:10.1101/pdb.prot5637.
- 37. Li, S.; Lefranc, M.P.; Miles, J.J.; Alamyar, E.; Giudicelli, V.; Duroux, P.; Freeman, J.D.; Corbin, V.D.A.; Scheerlinck, J.P.; Frohman, M.A.; Cameron, P.U.; Plebanski, M.; Loveland, B.; Burrows, S.R.; Papenfuss, A.T.; Gowans, E.J. IMGT/HighV QUEST paradigm for T cell receptor IMGT clonotype diversity and next generation repertoire immunoprofiling. Nature Communications 2013, 4, 2333. doi:10.1038/ncomms3333.
