# Supplementary material for: IMGT® Biocuration and Comparative Analysis of Bos taurus and Ovis aries TRA/TRD Loci
Source: Genes (Basel). 2020 Dec 28;12(1):30. doi: 10.3390/genes12010030 (PMC7824213; doi:10.3390/genes12010030)
Supplement: Supplementary file 1 [file genes-12-00030-s001.zip › Reviewer1_SK_161220.docx]

We thank the reviewer for having taken the time to go carefully through our manuscript. We acknowledge the constructive criticism and we address below the raised points.

This manuscript describes a new analysis of the TCR alpha and delta loci of the bovine and the sheep, and as such will be of interest to the veterinary immunology community in particular.

This is exactly our intention.

In its present form, it does not fully and adequately outline the changes that have arisen from this work. The manuscript should be an opportunity to bring clarity to our understanding of the loci of these species. In its current form it fails to do this.

We have taken into account several of the reviewer’s suggestions, we have modified the manuscript and we feel that the clarity of our understanding of the loci of the studied species has been considerably enhanced.

The Abstract gives undue attention to IMGT and its founding director. Almost half the abstract is given over to this background, which is irrelevant to the manuscript. These opening sentences have appeared almost word-for-word many times before. These sentences might be suitable in, for example, Lefranc 2015 (reference 1) and Lefranc 2014 (reference 2) which are historical accounts, but they are not suitable here. Given the word limit of an abstract, it is no surprise that with the few remaining words, the authors are unable to adequately describe the manuscript.

We took into account the constructive criticism of the reviewer and restructured the abstract.

‘IMGT’ is included as a Keyword. It is inappropriate for the name of a research group to be a Keyword.

We think that IMGT has got its place in the keywords as all the standards of the biocuration of the TR are based on the rules put forward by IMGT.

The attention to IMGT continues in the opening paragraph. The manuscript describes the TCR alpha and delta loci of the bovine and the sheep, and this should be the focus of the opening sentences of the Introduction.

The reviewer’s comment has been taken into account and the manuscript modified accordingly, lines 30-35.

The expression “IMGT 5’ borne” is a strange one, though a weblink is provided to an IMGT webpage which makes its meaning clear. Whether or not this is a useful term remains to be seen, though this reviewer cannot find an example of its use outside the publications of this group. Why is the name of the research group included in the term?

We acknowledge that giving the definition of this relatively new standard within the manuscript would have been appropriate. As we are talking about a standard, the IMGT prefix is used. We modified the manuscript accordingly, lines 96-107.

Why are these delimiters of the loci given such prominence in the Introduction, when almost nothing is said in the Introduction about the species under consideration?

Following the reviewer’s comment, information about the importance of the studied species was added in the manuscript, lines 64-67.

The value of a focus on these species is also poorly justified. There are many arguments for an interest in these species, and their use as models for coronavirus and influenza virus infections must be low in any ranking of those arguments. The Introduction should give much more attention to outlining the work that has been done on TCR genes in these species. In particular, references 8 to 14 each deserve a comprehensive outline and critique, and the context in which the new genome assemblies have arisen should be thoroughly described.

We thank the reviewer for the constructive criticism about the genome assemblies and we acknowledge the importance of discussing the quality of them. The information has been revisited and integrated in the manuscript, lines 72-77.

Although the methods used might be well explained by cited references, readers should not have to turn to other publications to gain a reasonable understanding of the methods. Lines 63-68, for example, give no idea of the most critical methods in the study. They simply invoke the 'authority' of the authors. The mention of the determination of functionality (lines 80-82) also gives no actual information on the methods used. The methods should be both described and justified. Other aspects of the Methods describe actions that generate information for IMGT, and the generated data may be provided at the IMGT website. Those data and outputs are not a part of the manuscript. An obvious example is the inclusion of the description of Colliers de Perles representations. Although these representations may be available at the IMGT website, it is not the purpose of a scientific report to advertise work that is not presented in the report.

The methodology described in detail was recently published in (Pegorier et al. 2020). We added the reference to this article and we provided within the manuscript a paragraph elaborating further the specificities pertaining to the under question study, lines 96-107.

Further details of the current assemblies and of previous assemblies should be provided. Are the new assemblies based upon resequencing of previous material, or a reinvestigation of the loci using samples from new animals? Are the breeds of the animals known?

We thank the reviewer for pointing out this important detail. The information has been added in the Introduction of the manuscript.

A major focus of the manuscript is comparisons. However critical information is missing or hard to find. To gain a better understanding, this reviewer turned to the IMGT website where it is clearly shown that since early 2020, huge changes have been made to the IMGT TRA and TRD Reference Directories for these two species. Surely this manuscript should document these changes, and should explain the reasoning behind the changes. There has been wholesale renaming of TRAV and TRAJ genes in particular, for both species. Why? ‘Functionality’ has changed for many sequences. Why? The details are important, and this is why the methods section needs to properly describe the way that functionality is determined. There has also been ‘updating of numbering’. Why?  Does ‘updating of reference sequences’ mean that the actual sequences have changed? Is this because of errors in previous sequences? These issues should be central to the manuscript.

The reviewer raises interesting questions. The information has been added in the Results section.

It is said (line 132) that the assemblies used in this study are better than previous assemblies. This is an issue of great importance, and is worthy of more detailed presentation.

We thank the reviewer for pointing out this indeed interesting point. The information is added in the manuscript and we discuss the criteria of the IMGT biocuration team to assess the quality of the different assemblies, lines 190-197.

What led to the differences? Why are the current assemblies better? Even if they are better, how likely is it that the current assemblies are accurate? What are the implications if the current assemblies are not accurate? If the new assemblies are from different animals, does this point to variation between bovine and ovine breeds, or perhaps to variation within breeds? What are the implications of this for the work that is presented? Do the different assemblies point to structural variation within the loci of these species? What would this mean for the analysis of the assembly that is described in this manuscript, and for the names that seem to have been assigned to genes as a consequence of the analysis?

The above questions are of great importance; however, they are out of the scope of the current manuscript.

Table 7 clearly shows that multiple alleles have been identified in both bovine and ovine. This requires explanation. It raises the question of what presented data comes from the new assembly, and what data comes from elsewhere. Variation between animals is a topic that needs to be openly addressed, and if it is well covered, it is a topic that would give even greater importance to the manuscript.

We thank the reviewer for this point. We added information on the precise number of the new alleles and we point out the names of them too.

Section 3.6 describes an analysis of cDNA sequences, but no details are provided. How many cDNA sequences were analyzed? What studies generated the sequences? What percentage of the germline genes were identified in the dataset? Did these sequences support prior determinations of functionality in all cases other than those mentioned in the text (involving stop codons)? TRAJ26 when expressed is described as ‘mutated’. What exactly does this mean? Is some special biological process being suggested? Similarly expressed TRDV1-17 is said to include deletions. Is some biological process being suggested here too?

Concerning the cDNA sequence analysis, additional information was added, lines 274-287. Concerning the potential biological processes involved, we prefer not to make any speculations that risk to cause more confusion to our readers than enhancing the understanding.

Line 228-230: What is meant here by ‘the expertise’?  The expression is unclear, but it seems like a kind of circular argument is being invoked. The ill-defined pipeline is giving rise to a dataset that is valued and correct because it is the product of the pipeline. Rather, the data that is generated from the pipeline should be adequately described and explored, so that readers can try to assess the data quality, and so that the research community can be better informed about the likelihood of problems with the pipeline. IMGT cannot rest on their laurels and simply claim that their pipeline is good because it is their pipeline and has been used before.

The methodology was described in detail in (Pegorier et al. 2020). More details were added in this manuscript too, section Materials & Methods

The conclusions as stated in the final sentence of the Discussion are inappropriate. In the absence of some kind of benchmarking, it is impossible to conclude whether or not the assemblies and annotations are accurate for the TCR loci. Even if tentative conclusions might be drawn with respect to TCR loci, the relevance of this to the IG loci is not discussed and arguably is not established.

Taking the reviewer’s point into account, we made more precise our conclusions, lines 304-309.

Self-citations: It is inevitable that many IMGT works will be cited in this study, but the number is excessive, and as outlined above, many are irrelevant to the study. The manuscript would be strengthened if independent non-IMGT analysis confirmed some of the IMGT processes and some of the IMGT findings that underpin this work.

The references have been revisited and adjusted taking into account the reviewer’s comments.

Minor issues:

- The TCR abbreviation is shortened in the manuscript to TR. This is confusing.

We are aware of several colleagues using the TCR abbreviation, however since the beginning of IMGT, more than 30 years ago, the abbreviation TR has been used and we feel strong about keeping it.

- Table 8 TRAV3 shows a difference that is not highlighted in red. The fact that the three numbers shown at each entry correspond to CDR1, CDR2 and CDR3 should be stated.

We thank the reviewer for the comment and we agree that further clarity is given by having implemented it in Table 8.

- Line 97: What are ‘expertised data’?

Given the added information of the methodology used from the biocuration team, we feel that this point is currently clearly addressed.

- Table 10 human TRDV1 shows a difference that is not highlighted in red. The colour system needs to be explained in the footnotes for each table where this is used. Again, the fact that the three numbers shown at each entry correspond to CDR1, CDR2 and CDR3 should be stated.

The color scheme has been revisited in Table 10 to add clarity.

- Line 235: What is the meaning of ‘fettering of genes in the locus’?

The sentence has been revisited to add clarity, lines 298-303.

- Line 241-241: The meaning of this sentence is unclear.

Clarity has been added, by rephrasing the sentence, lines 304-309.
