# Supplementary material for: IMGT® Biocuration and Comparative Analysis of Bos taurus and Ovis aries TRA/TRD Loci
Source: Genes (Basel). 2020 Dec 28;12(1):30. doi: 10.3390/genes12010030 (PMC7824213; doi:10.3390/genes12010030)
Supplement: Supplementary file 1 [file genes-12-00030-s001.zip › Reviewer2_SK_161220.docx]

The manuscript by Pegorier et al. provides a description of the TCRA/D locus in cow and sheep based on new genome assemblies and applies the IMGT nomenclature and annotation to these loci.  It is of value to those interested in cow and sheep immunology and the evolution of TCR genes.  The value of the manuscript itself is somewhat limited as most investigators will access this information directly at the IMGT website.

General comments:

The Abstract is not really an abstract of the work presented.  It is more a description of the IMGT program and almost no summary of what was actually presented in the paper.  Abstracts should summarize the findings in the paper and the one presented here should be revised.

We thank the reviewer for the constructive criticism and we provide a revised Abstract having taken into consideration the comments.

The term “IMGT borne” is an odd term that the IMGT database uses to describe non-IG or non-TR genes with conserved synteny between species.  If the authors wish to use this term it should be defined at the time of first use.  In the opinion of this reviewer, it is not a commonly used terminology in the field.

Lines 96-107 in the manuscript have been added to address the reviewer’s comment.

The authors should provide a better definition of how they are distinguishing between “functional” and “open reading frames”.  Is functional mean found being used and ORF one that looks functional but has not been found used in the repertoire? In the methods section they point to the IMGT website as the place to find out how they are defining the terms.  It would be helpful to have this defined in the paper than expect the reader to keep referring to a website for the information.  Why bother publishing the paper if the information is elsewhere online?

Taken into account the reviewer’s comment, the Methods section has been revised accordingly. Reported changes can be clearly tracked down in the manuscript, lines 127-132.
